# Structural transitions in Orb2 prion-like domain relevant for functional aggregation in memory consolidation

**DOI:** 10.1074/jbc.RA120.015211

**Published:** 2021-01-13

**Authors:** Javier Oroz, Sara S. Félix, Eurico J. Cabrita, Douglas V. Laurents

**Affiliations:** 1 Instituto de Química-Física Rocasolano, IQFR-CSIC, Madrid, Spain; 2 Departamento de Química Faculdade de Ciências e Tecnologia, UCIBIO, Universidade Nova de Lisboa, Caparica, Portugal

**Keywords:** nuclear magnetic resonance, NMR, memory consolidation, functional amyloid, prion-like domain, amyloidogenic proteins, amyloid, intrinsically disordered protein, Drosophila, metal ion-protein interaction, zinc, polyglutamine, pH regulation

## Abstract

The recent structural elucidation of *ex vivo Drosophila* Orb2 fibrils revealed a novel amyloid formed by interdigitated Gln and His residue side chains belonging to the prion-like domain. However, atomic-level details on the conformational transitions associated with memory consolidation remain unknown. Here, we have characterized the nascent conformation and dynamics of the prion-like domain (PLD) of Orb2A using a nonconventional liquid-state NMR spectroscopy strategy based on ^13^C detection to afford an essentially complete set of ^13^Cα, ^13^Cβ, ^1^Hα, and backbone ^13^CO and ^15^N assignments. At pH 4, where His residues are protonated, the PLD is disordered and flexible, except for a partially populated α-helix spanning residues 55–60, and binds RNA oligos, but not divalent cations. At pH 7, in contrast, His residues are predominantly neutral, and the Q/H segments adopt minor populations of helical structure, show decreased mobility and start to self-associate. At pH 7, the His residues do not bind RNA or Ca^2+^, but do bind Zn^2+^, which promotes further association. These findings represent a remarkable case of structural plasticity, based on which an updated model for Orb2A functional amyloidogenesis is suggested.

The comprehension of how the nervous system encodes memory has been an important goal since the beginning of modern neuroscience. At the turn of the twentieth century, memory was proposed to be encoded as alterations in the neurite network (1) as a persistent chemical change (2). This hypothesis has gained support over the decades (3, 4, 5, 6), and in 2003, Si *et al.* (7) published a seminal paper providing the first evidence for the molecular basis of memory consolidation. In that study, Si and co-workers proposed that functional aggregation of the protein CPEB into ordered structures is key to memory consolidation in *Aplysia*. Numerous studies have corroborated the key functional role of CPEB aggregation as well as that of its homologs in memory consolidation in *Drosophila melanogaster* and mammals (8).

The *Drosophila* CPEB homolog, named Orb2, has been investigated in the most detail. Two Orb2 isoforms, called Orb2A and Orb2B, are relevant for long-term memory in *Drosophila* (9, 10). Although they differ at the N terminus, both Orb2A and Orb2B contain a prion-like domain (PLD) rich in Gln and His residues, followed by a stretch of Gly and Ser residues (Fig. S1A). Although the Orb2A isoform is rare, it is essential for triggering the aggregation of the more abundant Orb2B and to maintain the memory trace (10, 11). The first nine residues of Orb2A are unique to this isoform (Fig. S1A). Rich in hydrophobic side chains, these nine residues have been reported to interact with membrane mimetics (12), to be crucial for aggregation (11), and to adopt a β-strand conformation within an Orb2 amyloid structure formed *in vitro* as characterized by solid-state NMR and EPR (13).

Both Orb2A and Orb2B contain a 31-residue-long Q/H-rich stretch, followed by a short amphiphilic segment (residues N_55_LSAL_59_) and a second modest Q/H-stretch (H_60_HHHQQQQQ_68_) (Fig. S1A). Because of its similarity to amyloid-forming polyQ stretches in huntingtin (14) and the androgen receptor (15), the main Q/H-rich stretch (residues 23–53) was suspected to be key for Orb2 amyloid formation. Indeed, while both Q/H-rich stretches appear rather disordered in Orb2 amyloids formed *in vitro* (13), Orb2 aggregation and memory consolidation can be blocked in *Drosophila* by a peptide which inhibits polyglutamine aggregation (16). Very recently, the elucidation of the structure of physiological Orb2 amyloid isolated directly from *Drosophila* fly brains has shown that the main Q/H-rich stretch does in fact form the physiologically relevant amyloid structure (17). This amyloid features 3-fold symmetry and side chain to main chain H-bonds (Fig. S1B). The participation of numerous His residues in the amyloid core provides a singular mechanism for amyloid destabilization through acidification. Intriguingly, the first nine residues of Orb2A, although relevant for triggering aggregation (11), do not contribute to the amyloid core, which is composed of the more abundant Orb2B isoform (17) (Fig. S1).

Following the second Q/H-rich stretch, there is a 200-residue, presumptively disordered region with an elevated content of Gly, Ser, Pro, and Asn (Figs. S1A and S2). The conserved C-terminal half of Orb2 is composed of two RRM domains, which bind Orb2-specific mRNA targets, and a ZZ-type zinc finger domain (Fig. S1A). ZZ-type zinc fingers are a special subclass that binds two Zn^2+^ ions and generally mediate protein/protein interactions, not nucleic acid binding (18). In Orb2, the conserved C-terminal domains are crucial for binding mRNAs containing a uridine-rich 3′ UTR, whose consensus sequence is ^5′^UUUUAU^3′^ (19), and potentially different translation complexes (17). This binding suppresses the translation of mRNAs coding for factors promoting synapse formation, synapse growth, and proteases (19). Upon neuronal stimulation, Orb2 forms amyloid aggregates leading to the activation of these mRNAs (20). Because of its physiological importance, Orb2 aggregation is finely controlled. Studies have reported key roles of intron retention (21), phosphorylation and protein stability (22), and chaperones (23) in tightly regulating Orb2 functional amyloid formation. Besides the mentioned control of Orb2 aggregation via acidification (17), Siemer and co-workers proposed that the spacing of His residues within the long Q/H-rich stretch would position them on the same face of a hypothetical α-helix (24). This may contribute to the binding of Ni^2+^, Cu^2+^, and Zn^2+^ which might impact aggregation (24). Interestingly, the longer Q/H-rich region of Orb2 may be sequestered into pathological amyloids formed by expanded polyQ segments of huntingtin, providing a novel explanation for memory loss in dementia (16, 25).

Whereas all these results have provided molecular insight into Orb2's role in memory consolidation, much is still unclear regarding the atomic-level details of the amyloid formation process. In particular, it is unknown whether the nascent protein contains elements of partial structure which predispose the formation of the functional amyloid, or alternatively, act as a safety mechanism to prevent premature or excess amyloid formation. The objective of this study is to characterize the atomic-level conformation and dynamics of Orb2A PLD and the first residues of the G/S-rich region using solution NMR spectroscopy. Because pH is proposed to impact amyloid formation and stability (17), we have characterized the conformation of Orb2A both at pH 4, where His residues are positively charged, which impedes aggregation, as well as pH 7, where neutral His are compatible with amyloid formation. In addition, we have probed the interaction of RNA ligands, Ca^2+^ and Zn^2+^ with the Q/H-rich regions of Orb2A. The structural transitions observed may represent nascent structures in the process of aggregation into functional amyloids.

## Results

### The Orb2A PLD is disordered at pH 4.0, except for a partly populated α-helix spanning residues 55–60

Initial 1D ^1^H and 2D ^1^H-^15^N HSQC spectra recorded in PBS at pH 7.0 revealed broad lines, which are not optimal for spectral analysis and assignment. Therefore, following the low pH, low ionic strength strategy of Song and coworkers (26), which we have recently applied to assign the snow flea antifreeze protein (27) and a RepA winged helix domain (28), spectra of Orb2A PLD were recorded at pH 4.0 in 1 mm acetic acid buffer at 25°C. Under these conditions, the signals were sharper and gave excellent quality 2D and 3D spectra, despite significant peak overlap. The ^1^Hα, ^1^HN, ^13^Cα, ^13^Cβ, and backbone ^15^N and ^13^CO chemical shifts at pH 4.0, 25°C, obtained through an unconventional strategy of proton-less 3D NMR spectra (see “Experimental procedures”), have been deposited in the BMRB data bank under accession number 50274. The assignments are complete except for the ^1^Hα of proline residues and the ^13^CO and ^13^Cβ of terminal Ser-88.

**Figure 1:**
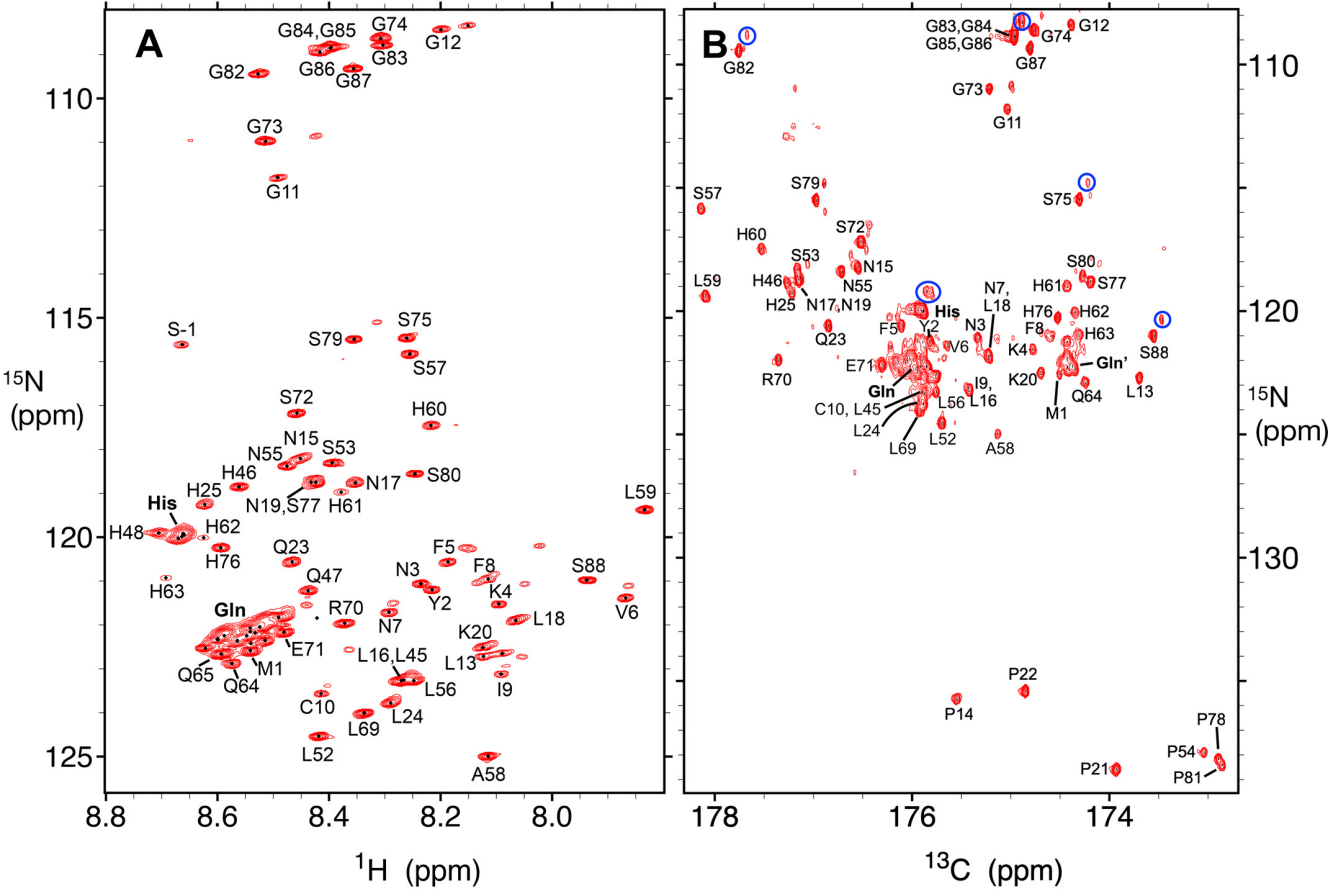
**Assigned 2D ^1^H-^15^N HSQC and 2D ^13^C-^15^N CON spectra of Orb2A PLD recorded at pH 4.0, 25°C.***A*, 2D ^1^H-^15^N HSQC spectrum of Orb2A PLD recorded at pH 4.0, 25 °C. Individual assigned signals are labeled. *S-1* corresponds to the Ser residue resulting from cloning and tag cleavage that precedes *M1*. The *bold* His and Gln labels refer to overlapped His and Gln peaks; these arise from residues in the amyloidogenic segment. The multiplication factor between contour levels is 1.4 here and in *panel B* as well as Fig. 4*A*. *B*, 2D ^13^C-^15^N CON spectrum of Orb2A PLD recorded at pH 4.0, 25°C. The labels shown correspond to the ^15^N signals; the ^13^CO correlations arise from the preceding residue. Note that the *y* axis scale is longer in *panel B* than *A* to include Pro residues. The *bold* His, Gln, and Gln′ labels are for overlapped His and Gln residues; the Gln′ cluster comes from *i* His ^13^CO/*i* + *1* Gln ^15^N correlations. For intense or overlapped peaks, a satellite peak is often seen slightly to the right and above the main peak. These satellite peaks arise from deuteration and some examples are indicated with *blue circles*.

The assigned 2D ^1^H-^15^N HSQC and 2D ^13^C-^15^N CON spectra registered at pH 4.0, 25°C, are shown in Fig. 1, *A* and *B*, respectively. The resonances are generally sharp and well defined except for clusters of overlapped Gln and His residues belonging to the amyloidogenic tract (Fig. S1B). The low ^1^HN signal dispersion is a hallmark of disordered proteins and an assessment of the ^13^Cα, ^13^Cβ, ^13^CO, and ^15^N chemical shifts using the TALOS+ program confirmed the overall dearth of structure in the Orb2A PLD under these conditions. With regard to the glutamine side chain amide groups, their ^1^H_2_-^15^N signals are also clumped into overlapped peaks (Fig. S3). Notably, they do not show the striking pattern of disperse signals observed for the glutamine side chain resonances of the androgen receptor, which participate in side chain to backbone hydrogen bonds (29). Orb2A PLD contains a single Cys residue. Its ^13^Cβ chemical shift indicates that it is reduced at pH 4.0 in 1 mm acetic acid buffer in the absence of reducing agent. Regarding the proline residues, all Xaa-Pro peptide bonds are mainly in the *trans* conformation, which is expected for a disordered chain (30), as judged by their observed ^13^Cβ and ^13^CO chemical shifts.

**Figure 2:**
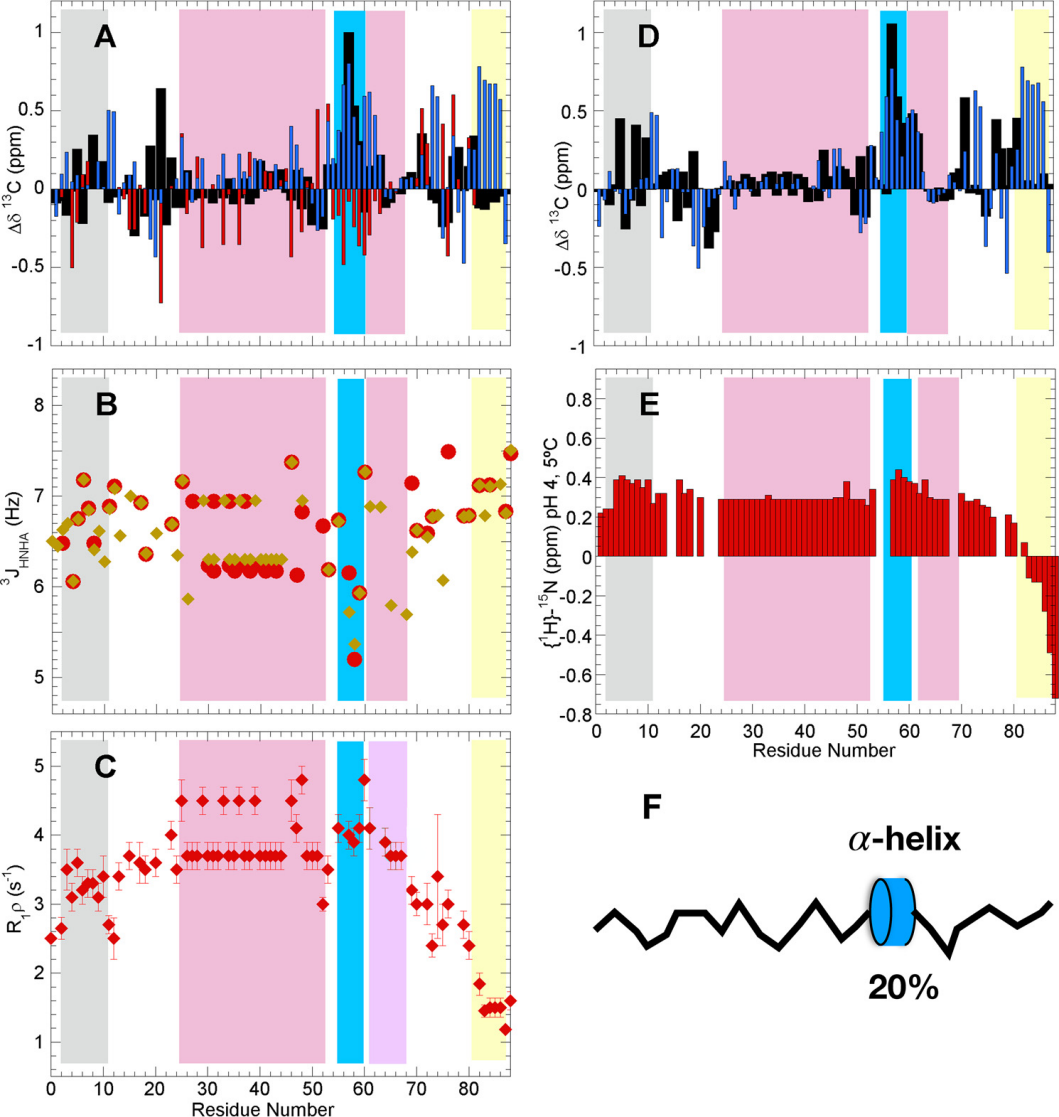
**Orb2A PLD is largely disordered at pH 4, save a short α-helix in residues 55–60.** In all panels, the different zones of the Orb2 PLD are shaded as follows: N-terminal hydrophobic stretch, *gray*; Q/H-rich, *cyan*; residues 55–60, *blue*; H·Q-rich, *cyan*, G-rich, *yellow*. In the Q/H or H·Q-rich regions, most of the values shown come from averaging of overlapped His or Gln peaks. *A*, conformational chemical shifts (Δδ) of Orb2 PLD at pH 4, 25°C. ^13^Cα, *black bars*; ^13^CO, *blue bars*; ^13^Cβ, *red bars*. Experimental uncertainties are ≤ ± 0.04, 0.02, and 0.08 ppm for ^13^Cα, ^13^CO, and ^13^Cβ, respectively. *B*, ^3^J_HNHA_ coupling constants at pH 4, 25°C. Values of 5 Hz or less are indicative of α-helix; higher values, coil or extended conformations. Values shown in *gold* were measured by peak height using the Sparky program. Values in *red* were obtained using the peak integral function of Topspin 4.0. *C*, transverse relaxation rates in the rotating frame (R_1_ρ) at pH 4.0, 25°C. *D*, conformational chemical shifts (Δδ) of Orb2 PLD at pH 4, 5°C. ^13^Cα, *black bars*; ^13^CO, *blue bars*. The experimental uncertainties are generally ≤ ± 0.05 ppm for both ^13^Cα and ^13^CO. *E*, {^1^H}-^15^N NOE at pH 4.0, 5.0°C. Values approaching 0.85 indicate stiffness on fast ps/ns and are typical of well-folded rigid proteins; values less than 0 indicate a high flexibility. Uncertainties are about ± 0.01. *F*, schematic diagram of Orb2 PLD at pH 4 featuring a flexible and disordered conformational ensemble with a short, modestly populated α-helix spanning residues 55–60.

The ^13^Cα, ^13^Cβ, and ^13^CO Δδ values, which reveal partially populated elements of secondary structure, are plotted in Fig. 2*A*. The segment composed of residues 55–60, which follows the amyloidogenic Q/H-rich stretch, adopts a minor population of α-helix. Based on the conformational chemical shift (Δδ) values expected for 100% α-helix of 3.1, −0.4 and 2.2 ppm for ^13^Cα, ^13^Cβ, and ^13^CO, respectively (31, 32), the population of α-helix for these residues is ∼20%. Otherwise, the N-terminal hydrophobic stretch, the Q/H-rich segments and the C-terminal G-rich element do not contain detectable populations of secondary structure under these conditions. Whereas the Δδ ^13^CO values of terminal G_82_GGGGG_87_ are anomalously high at pH 4 (and also pH 7, see below), which suggests α-helix formation, they are not corroborated by high Δδ ^13^Cα values, so this may rather reflect a shortcoming in the prediction of secondary structure from ^13^CO δ values for polyG repeats. As an orthogonal measure of secondary structure, the ^3^J_HNHA_ coupling constants were measured and analyzed using the Karplus equation to obtain backbone ϕ angles. Some ^3^J_HNHA_ coupling constants appear to be somewhat lower for residues 55–60 (Fig. 2*B*), which may reflect the minor population of α-helix. Next, the µs/ms dynamics were assessed by R_1_ρ relaxation measurements. The obtained values are consistent with high mobility, except for residues 55–60, whose slightly elevated R_1_ρ rates reflect a modestly increased rigidity (Fig. 2*C*). Therefore, the NMR data indicate formation of a small population of α-helical structure in residues 55–60 of the Orb2A PLD.

The population of marginally stable elements of secondary structure in polypeptides is generally enhanced by cooling. To check if this is the case for Orb2A PLD, additional spectra were recorded at 15°C and 5°C, pH 4.0. Nevertheless, the analysis of the conformational chemical shifts did not reveal any additional elements of secondary structure, beyond the 55-60 helix already detected at 25 °C (Fig. 2*D*). Next, the fast ps/ns dynamics were characterized by {^1^H}-^15^N NOE measurements at 5°C. Low values indicative of flexibility are seen throughout the domain and are only slightly higher for the hydrophobic segment and α-helix spanning residues 55–60 (Fig. 2*E*). The G/S-rich C-terminal residues are especially mobile. Finally, the presence of helical conformations by residues Asn-55–His-60 is further supported by the observation of HN_i_-HN_i+1_ NOES between Ala-58–Leu-59, Leu-59–His-60, and His-60–His-61 and Hβ_i_-HN_i+1_ NOEs between Asn-55–Leu-56, Leu-56–Ser-57, and Ala-58–Leu-59, in a 3D ^1^H-^1^H-^15^N NOESY·HSQC spectrum (Fig. S4). Overall, we can conclude that at pH 4.0, where the His residues of Orb2A's PLD are chiefly positively charged, the protein is disordered and flexible, except for the minor population of α-helix formed by residues 55–60 (Fig. 2*F*).

### The amyloidogenic Q/H-rich segment adopts partly populated helical conformations and rigidifies at neutral pH

**Figure 3:**
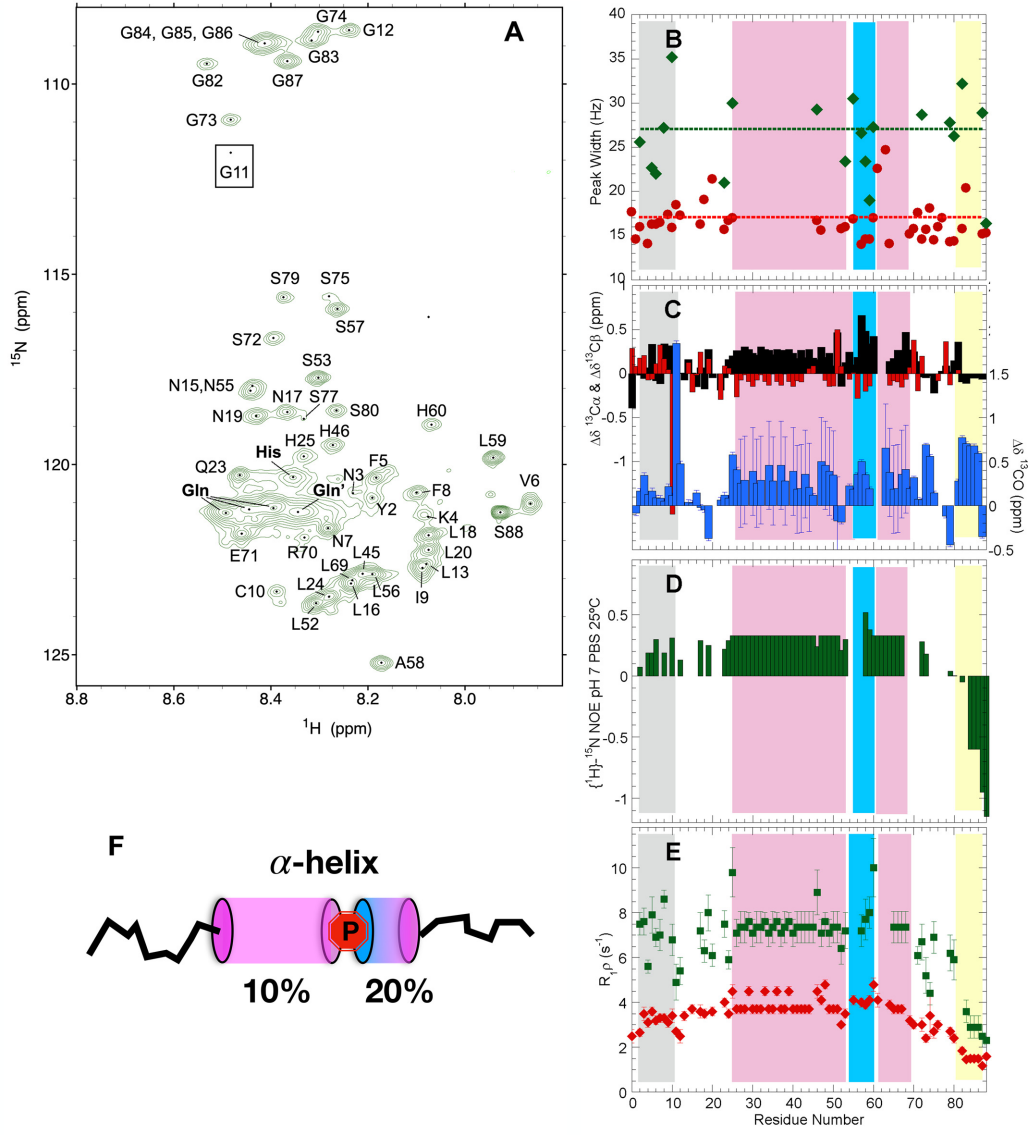
**The Q/H-stretch of Orb2A partially adopts α-helical conformations and rigidifies at pH 7.***B*–*E*, the different zones of the Orb2 PLD are shaded as follows: N-terminal hydrophobic stretch, *gray*; Q/H-rich, *light magenta*; residues 55–60, *blue*; H·Q-rich, *light magenta*; G-rich, *yellow*. In the Q/H or H·Q-rich regions most of the values shown come from averaging of overlapped His or Gln peaks. *A*, 2D ^1^H-^15^N HSQC spectrum of Orb2A PLD registered at pH 7.0, 25°C. The bold His, Gln, and Gln′ labels refer to overlapped His and Gln peaks; these arise from residues in the amyloidogenic segment. The Gln′ peak arises from Gln, which follows His along the sequence. The signal of Gly-11 (*boxed*) is beneath the lowest selected contour level. To afford a fair appreciation of the wider peak widths observed here relative to the spectrum at pH 4, the same axes, aspect ratio, and multiplication factor between contour levels (1.4) are used here as in Fig. 2*A*. *B*, ^1^H peak width of Orb2 PLD ^1^H-^15^N HSQC peaks at 25°C and pH 7 (*green diamonds*) and pH 4 (*red circles*). The *dotted lines* represent the average peak widths at pH 7 (*green*) and pH 4 (*red*). Overlapped peaks are excluded. The uncertainties are approximately ± 1.5 Hz. *C*, conformational chemical shifts for ^13^Cα (*black bars*), ^13^Cβ (*red bars*), and ^13^CO (*blue bars*) at pH 7.0, 25°C. For clarity, the *y* axis scale is shifted for the ^13^CO Δδ values. The ^13^CO of Gly-11 and the ^13^Cβ of Cys are outliers. The ^13^CO Δδ values of the Q-rich region have large uncertainties because of the broad nature of the overlapped peak of Q residues. *D*, fast ps/ns dynamics assessed by the heteronuclear {^1^H}-^15^N NOE. Uncertainties are about ± 0.015. *E*, transverse relaxation rates in the rotating frame (R_1_ρ) for Orb2 PLD at pH 7.0, 25°C (*green squares*). For comparison, the R_1_ρ value obtained at pH 4.0, 25°C and already shown in Fig. 3*C* are also shown here by *red diamonds*. *F*, at pH 7, the Q/H segments (*magenta cylinders*), and the 55–60 segment (*blue cylinder*) adopts a small but detectable population of α-helical conformers separated by proline 54 (*red*).

A visible precipitate formed when the pH of the sample was increased from 4.0 to 7.0. Nevertheless, the concentration of protein remaining in solution was sufficient to record good quality 2D ^1^H-^15^N and ^1^H-^13^C HSQC as well as 3D ^1^H-detected HNCO, HNCA, and CBCAcoNH spectra. The 2D ^1^H-^15^N HSQC spectrum (Fig. 3*A*) is significantly broadened. The average ^1^HN peak width is 27 ± 4 Hz at pH 7.0 compared with 17 ± 2 Hz at pH 4.0 (Fig. 3*B*). This is an indication of conformational interconversion, oligomerization, or both (33). The assignments at pH 7.0 are complete for ^13^Cα, ^13^Cβ, ^1^HN, and backbone ^13^CO and ^15^N nuclei save the ^13^Cβ of Ser-77, the ^13^CO of Ser-79, the ^13^Cβ and ^13^CO of Ser-88, as well as the ^13^Cβ and ^13^CO of Leu-15 and other residues preceding proline residues. Moreover, all assignments are missing for His-63, His-64, and His-78 as these residues' ^1^HN signals may be adversely affected by exchange broadening. The assigned chemical shift values, like those measured at pH 4.0, have been deposited in the BMRB under accession number 50274.

Remarkably, the assessment of the ^13^Cα, ^13^Cβ, and ^13^CO Δδ values shows that at neutral pH, the main Q/H-rich stretch partly adopts α-helical conformations, whose population is ∼10% (Fig. 3*C*). Residues Ser-53–Pro-54 separate this helix from that formed by residues 55–60. The shorter Q/H segment also appears to adopt a minor population of α-helix. The nine N-terminal residues do not show a clear tendency to adopt a preferred secondary structure at pH 7. Cys-10 remains reduced and Xaa-Pro peptide bonds continue to be mainly *trans* at pH 7.

To corroborate the pH-dependent conformational changes, a series of 2D ^1^H-^15^N HSQC spectra were recorded on a fresh ^15^N-labeled sample of Orb2A PLD, at 25°C and pH values ranging from 3.5 to 7.3 (Fig. S5A). Interestingly enough, the ^15^N conformational chemical shifts for residues in the Q/H-rich segments become more negative at pH values where His residues are neutral (Fig. S5B), which suggests an increase in α-helical conformers (34). This observation is consistent with the increase in α-helical structure detected by the Δδ^13^Cα, Δδ^13^Cβ, and Δδ^13^CO values (Fig. 3*C*). By contrast, the Δδ^15^N values of Gly residues near the C terminus show small changes. Those of Ala-58 and Leu-59 suggest a decreased helicity at pH 7 (Fig. S5), which may be because of the loss of a favorable charge/helix macrodipole interaction (35) when the His residues following Leu-59 lose their positive charge.

Relaxation measurements at pH 7 yielded {^1^H}-^15^N NOE and R_1_ρ values that are elevated for most residues (Fig. 3, *D* and *E*) relative to values obtained at pH 4.0 (Fig. 2, *C* and *E*). These measurements reflect stiffening on ps/ns and µs/ms time scales and are consistent with the formation of preferred conformers (Fig. 3*F*) and associative processes. However, the G/S-rich segment located C-terminal to the PLD continues to be highly dynamic at pH 7, both on faster ps/ns time scales as well as slower µs/ms time scales.

**Figure 4:**
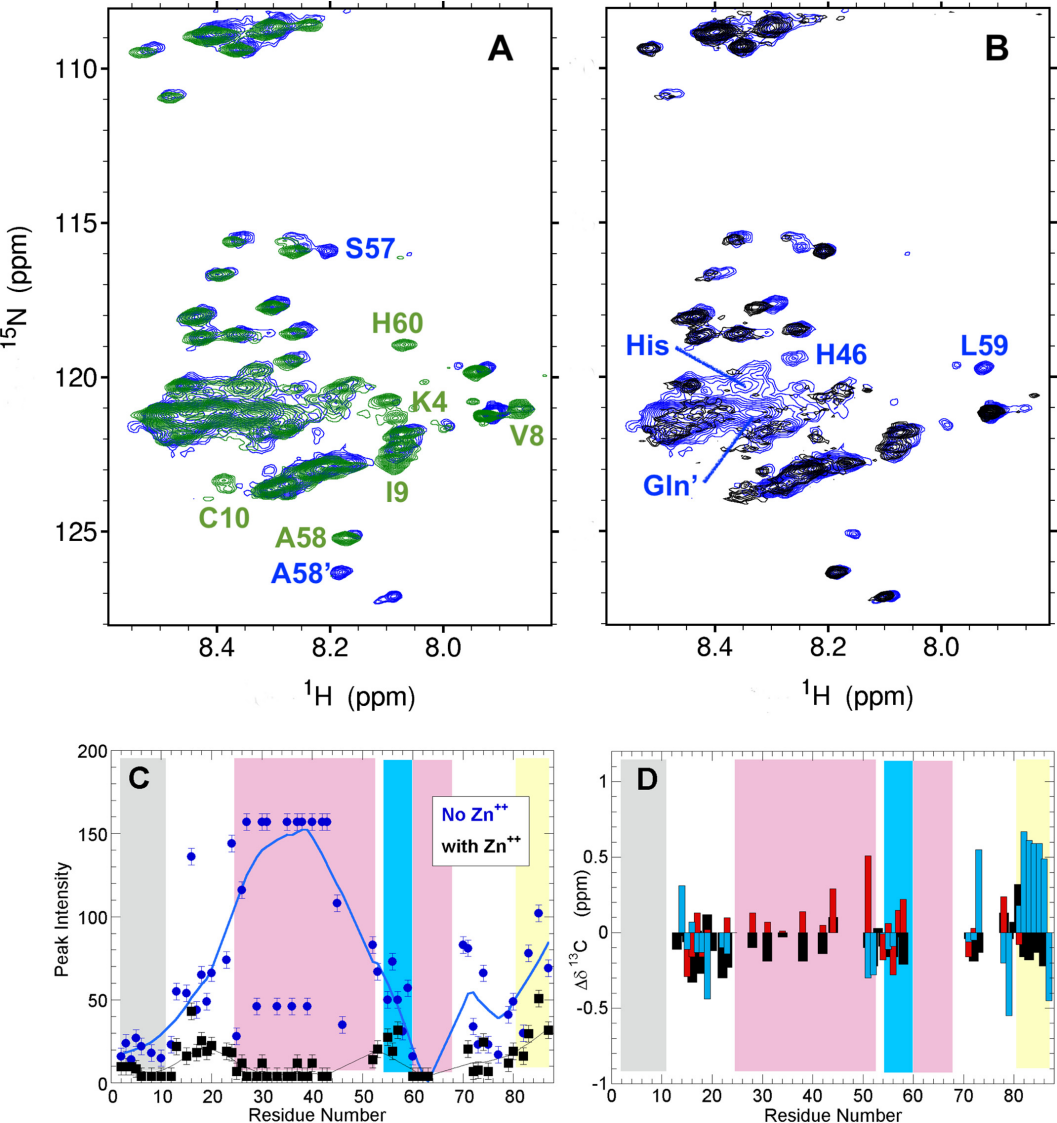
**Zn^2+^ binding to His residues at pH 7 promotes further oligomerization.***A*, ^1^H-^15^N HSQC before (*green*) and after (*blue*) the 2-month pause. Some distinct residues are labeled. *B*, ^1^H-^15^N before (*blue*) and after (*black*) the addition of ZnCl_2_. His and Gln signals (labeled) weaken sharply after addition of Zn^2+^. *C*, the largest drop in peak intensity upon Zn addition occurs for residues of the Q/H-rich segments (*rose band*). *D*, conformational chemical shifts for ^13^Cα (*black*), ^13^Cβ (*red*), and ^13^CO (*blue*) strongly suggest that the molecules remaining in solution lack helical zones and are disordered.

After a 2-month incubation at 4°C, Orb2A PLD's ^1^H-^15^N NMR spectra at 25°C in PBS revealed that despite some changes, most peaks retained their positions; this suggests that slow conformational changes or aggregative processes are minimal (Fig. 4*A*). Zinc chloride was then added to a final concentration of 2 mm. In the presence of this divalent cation, the peak intensities of His and Gln ^1^H-^15^N resonances drop practically to the signal/noise limit (Fig. 4, *B* and *C*). Strong decreases are also seen for the ^1^H-^13^Cβ and ^1^H-^13^C Cα signals of His in 2D ^1^H-^13^C HSQC spectra (Fig. S6). This strongly suggests that Zn^2+^ binds to His residues and promotes association processes of nearby Gln residues which are manifested as a loss of signal intensity. The signal intensity of other residues also decreased following Zn^2+^ addition, but sufficed to record good quality 3D HNCO, HNCA and CBCAcoNH spectra. The analysis of these spectra revealed that the conformational ensemble of the molecules remaining visible to NMR is poor in helical structures and slightly enriched in extended conformations (Fig. 4*D*). Thus, Zn^2+^ binds the His residues and drives the association of helical conformations of Orb2A PLD into large assemblies invisible to liquid state NMR.

### Orb2A PLD binding to Zn^2+^ and RNA oligos is pH dependent

To confirm that Zn^2+^ binds Orb2A PLD His residues, we performed H/D exchange to remove all ^1^HN signals, leaving the His, Phe, and Tyr aromatic ring ^1^H as the only signals in the downfield region. Because His ^1^Hδ2 and ^1^Hε1 lie close to the side chain nitrogen atom that putatively ligates Zn^2+^, their NMR signals should be sensitive probes of this binding. In addition to Zn^2+^, the entry of Ca^2+^ is important for nerve impulses. No changes in the His ^1^Hδ2 and ^1^Hε1 resonances were observed under acidic conditions (pH 4.0, 25°C), following the addition of a 10-fold excess of Ca^2+^ and Zn^2+^ (Fig. S7A). Therefore, we conclude that these cations do not bind to the His residues of Orb2A PLD when they are in the imidazolium form. The charged state of His residues may affect their capacity to bind Ca^2+^ and Zn^2+^. Upon titration with Ca^2+^ to a 10-fold excess relative to a fresh Orb2A PLD sample at neutral pH, 25°C, no visible alterations in the His ^1^Hδ2 and ^1^Hε1 signals were seen (Fig. S7B). In contrast, significant changes in these resonances were observed upon adding a 10-fold excess of Zn^2+^ at pH 7.0, 25°C.

Whereas the RRM and ZnF domains of Orb2 have been reported to bind to specific RNA sequences in the 3′ UTR (19) because of their charged nature at low pH, the Q/H-rich segments might also be able to bind RNA. To test this idea, 2D ^1^H-^15^N HSQC spectra of Orb2A PLD were recorded and show significant changes at pH 4 (where His residues are mostly cationic) in the presence of ^5′^UUUUUAU^3′^, the consensus ligand, as well as ^5′^CCCCCGC^3′^, the opposite sequence studied as a negative control (Fig. S8A). In particular, many peaks corresponding to residues near the N terminus and in the residues 55–60 α-helix disappear while those arising from residues in the Q/H-rich stretches shift and weaken. These changes are consistent with observation of visible aggregates upon mixing Orb2 PLD with the RNA oligos. However, residues beyond Arg-70 show much smaller shifts and remain relatively strong. This suggests that the Gly-rich C-terminal region does not participate in RNA binding or aggregation. The RNA-induced alterations in Orb2A PLD's HSQC spectra are much less striking at pH 7, where His residues are chiefly neutral (Fig. S8B). These observations lead us to suggest that the Orb2A PLD can bind RNA nonspecifically at pH 4 and that the binding is mostly mediated by electrostatic interactions.

## Discussion

The key findings of this study are that the amyloidogenic Q/H-rich region of Orb2's PLD is disordered and flexible at low pH but adopts a minor population of α-helix at pH 7.0, where the His residues are mostly in the neutral state. The hypothesis that Orb2 aggregation triggers enduring synapse-specific alterations in translation which are key for memory consolidation received strong support by the elucidation of its amyloid structure from adult *Drosophila* heads (17). Gln and His side chains are interlocked in this amyloid (Fig. S1B). The relative instability of Orb2 amyloid at acidic conditions, as shown in Fig. S15 of Ref. 17, can be attributed to His side chains becoming positively charged and producing electrostatic repulsion at low pH. *In vivo*, this may facilitate the disintegration of Orb2 amyloid fibrils in the lysosome. Considering the results reported here, is it possible that Orb2 PLD histidines could be initially maintained in a charged state *in vivo* and then neutralized to help trigger amyloid formation?

**Figure 5:**
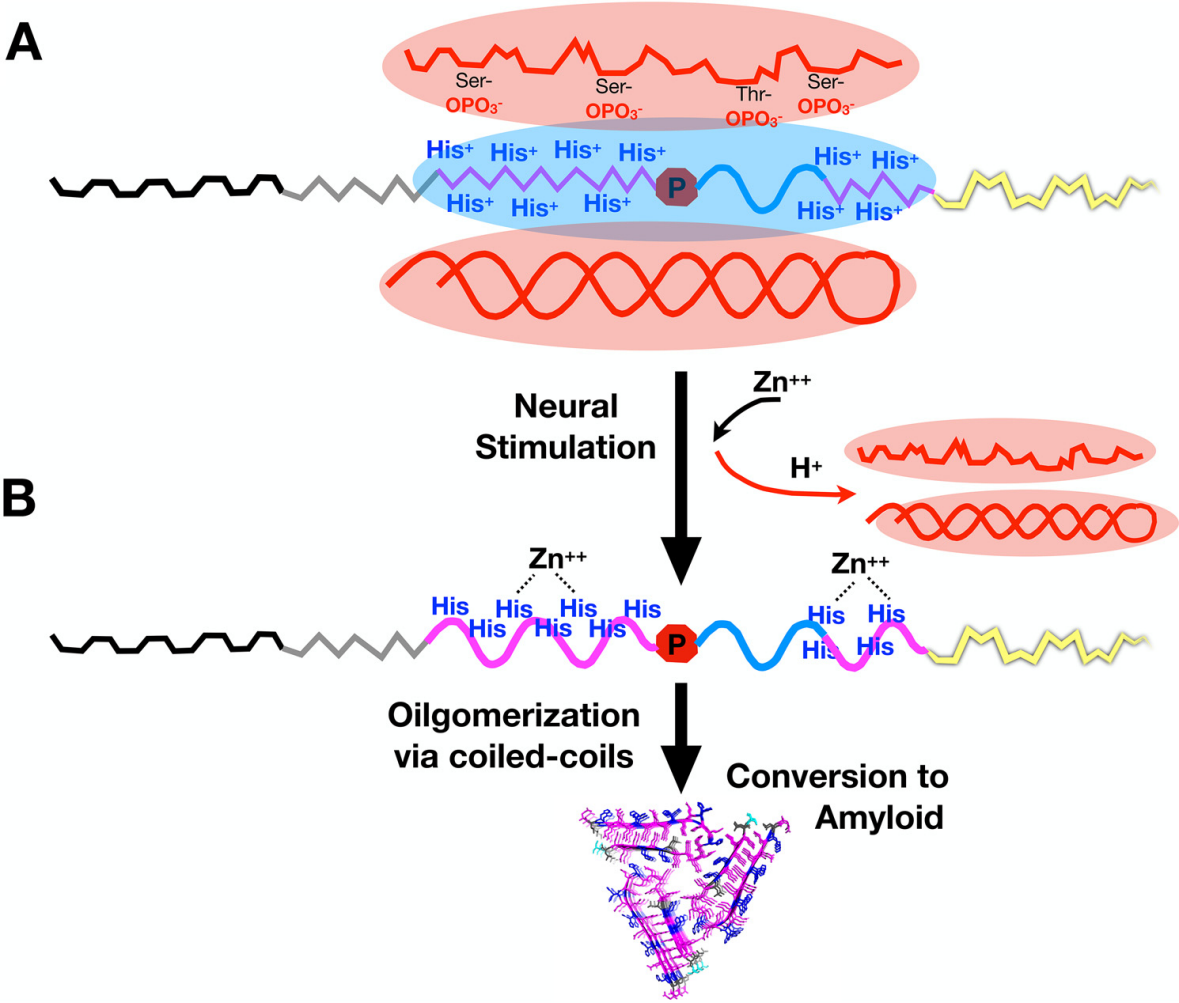
**His neutralization unleashes α-helix formation, oligomerization and amyloidogenesis.***A*, the first N-terminal hydrophobic segment (*black*) may interact with membranes which promote partial α-helix formation as reported by Soria *et al.* (12). The proximity of phosphorylated Tob protein (*top*, *red*), RNA (*bottom*, *red*), and/or possibly other polyanions, creates a negatively charged milieu which favors the cationic form of the 12 His residues (His +) of the Q/H-rich segments (*magenta*). This keeps the Q/H-rich segments disordered. Pro-54 (*red hexagon*) would also act to prevent premature amyloidogenesis. The 55–60 residue segment (*blue*) adopts a partial α-helix whereas the G/S-rich segment (*yellow*) remains disordered and flexible. *B*, following neural stimulation, the entry of Zn^2+^ and other cations could displace H^+^ and bind to the neutral form of His residues (*blue*) as proposed by Bajakian *et al.* (24); releasing Tob and RNA (*red*). The neutral form of His allows partial α-helix formation of the Q/H-rich segments, unlocking pathways to amyloid formation through coiled-coil intermediates.

In the *Drosophila* synapse, Orb2 monomers are initially bound to target mRNA molecules via their RRM domains. The proximity of polyanionic mRNA, phosphorylated Tob (22), and/or possibly other anions of the neuronal granule (36) could tend to stabilize the charged imidazolium form of His side chains. This possibility is supported by our observations that the PLD of Orb2 can bind to RNA under acidic conditions (Fig. S8). Upon neural stimulation, the influx of Ca^2+^ and Zn^2+^ cations could displace the imidazolium H^+^ and bind neutral His. This scenario is supported by ITC measurements demonstrating that the Q/H-rich region of Orb2 binds transition metal cations like Zn^2+^, but not Ca^2+^ (24), which we corroborate here by NMR (Fig. 4 and Figs. S6 and S7). Those researchers also proposed that if this segment were to adopt an α-helical conformation (just as we have shown here), then the His residues, which are mostly spaced *i, i* + *3* and *i, i* + *4*, would be mainly positioned on the same side of the α-helix (24). The binding of cations such as Zn^2+^ to such an array of His residues could increase the formation of α-helix, although this point is difficult to test because of the Orb2A PLD aggregation that accompanies cation binding, even at the low concentrations used for CD spectroscopy (24). The resulting helical conformations may drive association processes such as the formation of coiled coils, as seen for other proteins which undergo liquid-liquid phase separation via intermolecular helical associations (37). Q-rich coiled coils and higher oligomeric species have been proposed as intermediates in amyloidogenesis of *Aplysia* and mammalian CPEBs (38, 39, 40, 41), which are probably heterogeneous as strongly suggested by kinetic modeling (42). A hypothetical working model that incorporates these ideas is shown in Fig. 5.

The role of the initial hydrophobic segment (Fig. 1*A*) in Orb2A on amyloidogenesis has been debated. It was found to be important for Orb2-mediated memory, as even the conservative substitution of Phe-5 for Tyr affected activity (11). Moreover, this segment, and not the amyloidogenic Q/H-rich stretch, was found to adopt an α-helix that later evolved into amyloid *in vitro* as monitored by circular dichorism spectroscopy and solid state NMR spectroscopy (13). However, this segment was not observed in the physiological amyloid, which is composed chiefly of Orb2B, formed in *Drosophila* heads (17). Instead, the amyloid is composed of the longer Q/H-rich stretch (Fig. S1). Here, this Orb2A N-terminal hydrophobic segment was found to be devoid of detectable populations of secondary structure, at both pH 4.0 and pH 7.0. By contrast, this segment was reported to form an amphiphilic α-helix in the presence of membrane mimetics (12). Therefore, it is possible that under *in vivo* conditions, this segment may form an α-helix that facilitates functional amyloid formation.

Although *Aplysia* and mammalian CPEB homologs share a similar domain organization with *Drosophila* Orb2, they lack the multiple His residues interspersed in their Q-rich segments (40, 43, 44), which are found in Orb2. This suggests that the pH-regulated nature of Orb2 amyloid formation and dissociation may be substituted by other mechanisms. Proline 54, which interrupts the helices formed by the Q/H-rich segments and residues 55–60, may play an important role in preventing premature amyloid formation. Similar proline residues that delimit α-helices have been recently described in human CPEB3 (41).

Overall, our observations reveal remarkable structural plasticity in the region of Orb2A which is key for amyloid formation. The characterization of nascent Orb2A conformers presented here, together with the recent structural elucidation of Orb2 native amyloid fibrils (17), provides the cadre for understanding Orb2 amyloidogenesis. Further investigation is required to fully comprehend this relevant process.

## Experimental procedures

DNA coding for the first 88 residues of Orb2A is M_1_YNKFV-NFIC_10_-GGLPNLNLNK_20_-PPQLHQQQHQ_30_-QQHQQHQQH-Q_40_-QQQQLHQHQQ_50_-QLSPNLSALH_60_-HHHQQQQQ*LR_70_*-*ESGGSHSPSS_80_-PGGGGGGS_88_*, which comprises the PLD domain (*underlined*) and initial residues of the Gly/Ser-rich region (shown in *italics* above), was subcloned into a pET28(a) vector containing a TXA fusion protein, a His_6_ tag for IMAC purification and a TEV protease tag. After TEV cleavage, Gly and Ser residues remained attached to the N terminus of the Orb2A protein construct. Following expression in minimal media enriched with ^15^NH_4_Cl or ^15^NH_4_Cl and ^13^C-glucose as the sole sources of nitrogen and carbon, the resulting ^15^N- or ^15^N,^13^C-labeled protein was purified by Ni^2+^ affinity chromatography using a GE Biosciences ÄKTA FPLC system, using the buffer 20 mm Tris HCl, 500 mm NaCl, pH 8.0, adding imidazole (500 mm) for the elution. Next, the protein was cleaved with TEV protease, using 20 mm Tris HCl, 150 mm NaCl, 1 mm DTT, 0.5 mm EDTA, pH 7.0, and re-purified to remove the cut TXA and His tag. Because of the high number of His residues in Orb2A, the protein remained attached to the column and co-eluted with TXA. To remove the fusion protein, we performed anion exchange chromatography using 20 mm Tris HCl, 8 m urea, 1 mm DTT, pH 7.0, where the elution was achieved applying a linear gradient until 1 m NaCl was present in the buffer. The presence of 8 M urea prevents premature aggregation during purification and storage and is a common practice for amyloidogenic proteins, including the Orb2A (12, 13). The resulting protein is electrophoretically pure and was stored in concentrated urea at −80°C until use. Its purity and identity were subsequently corroborated by NMR spectroscopy.

Just prior to NMR spectroscopy, the urea was removed and the protein was transferred to 1.0 mm deuterated acetic acid buffer containing 15% D_2_O (99.96% atom D, Euroisotop), pH 4.0, using a PD-10 (GE Biosciences) desalting column. This concentration of D_2_O (15%) is somewhat higher than the 10% typically used; it was necessary to avoid losing the lock during the acquisition of long spectra. Following the renovation of the NMR consoles in April 2020, we recorded spectra with 10% D_2_O and obtained similar Orb2A spectra. By refractive index, NMR samples were found to contain less than 10 mm residual urea. This concentration of urea has a negligible effect on protein conformational stability (45). The concentration of Orb2A PLD samples was measured by UV absorbance using an extinction coefficient of 1490 cm^−1^·M^−1^ at 280 nm based on its content of one Tyr residue (46).

Following NMR spectral acquisition at pH 4.0, the sample was transferred to pH 7.0 by adding a 10× buffer stock of PBS prepared in D_2_O. Upon adding the PBS stock, the initial pH was 6.2; it was increased to 7.01 by adding small aliquots of 0.3 M KOH. The final buffer content of the pH 7 sample was 0.9 mm deuterated acetate, 25 mm Na_2_HPO_4_, 25 mm NaH_2_PO_4_, 100 mm KCl, and 1 mm TCEP as the reducing agent. The final sample volumes were 0.201 ml (pH 4) and 0.226 ml (pH 7). A final series of 2D and 3D spectra was recorded following the addition of ZnCl_2_ (Fluka) to a final concentration of 2 mm. All spectra were recorded in a water-matched, 5 mm diameter Shigemi NMR tube.

### NMR spectral assignments

All NMR spectra were recorded on a Bruker Avance NMR spectrometer operating at 800 MHz (^1^H), except a 3D ^1^H-^1^H-^15^N NOESY·HSQC spectrum and those examining the effects of pH, and the binding of RNA, Ca^2+^, and Zn^2+^, which were recorded with a Bruker 800 MHz Neo Avance NMR spectrometer. The spectrometer is equipped with a triple resonance (^1^H, ^13^C, ^15^N) cryoprobe and Z-gradients. The program Topspin (Bruker Biospin) versions 2.1 and 4.0.8 were used to record, process, and analyze the spectra. Because the Orb2A's N-terminal region is disordered and its sequence is of low complexity and contains many repeated consecutive residues, it presents special challenges for NMR spectral assignment. Therefore, instead of the standard approach based by ^1^HN excitation and ^13^Cα and ^13^Cβ connectivities (47), we have applied an unconventional proton-less approach based on a pair of 3D ^13^C-detected spectra which provide consecutive ^13^CO and ^15^N backbone connectivities as these nuclei retain more dispersion in IDPs (48). Even with this approach, some cases of sequential residues with identical ^13^CO and ^15^N chemical shift values were observed. Therefore, to overcome these ambiguities, an additional 3D ^1^H-detected spectrum that yields consecutive ^1^HN-^1^HN connectivities was recorded (49, 50). The analysis of these data led to the essentially complete spectral assignment at pH 4.0 as described in “Results.” After measuring relaxation measurements at pH 4.0, the sample pH was increased to pH 7.0. As described “Results,” the sample tends to aggregate at neutral pH. Because of its lower solution concentration, the assignment was carried out by conventional ^1^H-detected 2D ^1^H-^15^N HSQC and 3D CBCAcoNH, HNCO and HNCA spectra, and comparison with the pH 4 spectra. A complete list of the NMR experiments and their parameters is shown in Table S1. ^1^H peak widths in ^1^H-^15^N HSQC spectra were measured using a Gaussian function in the program NMRFAM Sparky 1.4. ^3^J_HNHA_ coupling constants were calculated based on the relative signal intensities of the ^1^HN/^1^Hα crosspeak: ^1^HN/^1^HN diagonal peak using either peak integration in Topspin 4.0 or peak heights in NMRFAM Sparky 1.4 as described previously (51).

The expected chemical shift values for the ^13^Cα, ^13^Cβ, ^1^Hα, ^1^HN, ^15^N, and ^13^CO nuclei in statistical coil ensembles were calculated using the parameters tabulated by Kjaergaard and co-workers (52, 53) and implemented on the RRID:SCR_019189 server at the Bax laboratory. These values were subtracted from the experimentally measured chemical shift values (δ) to calculate conformational chemical shifts (Δδ).

##### 3D ^1^H-^1^H-^15^N NOESY·HSQC spectrum.

To obtain additional evidence for secondary structure formation, a 3D ^1^H-^1^H-^15^N NOESY·HSQC spectrum was recorded at 25°C, pH 4.10, with a 100 ms mixing time. The sample contained 1 mm deuterated acetic acid and 1.17 mm^15^N-labeled Orb2A PLD. Because of severe signal overlap, we forwent analysis of the Q/H-rich stretches and focused on residues 55–60.

##### pH titration followed by 2D ^1^H-^15^N HSQC spectroscopy.

Further testing of the assignments and helical structure was performed by recording a series of five 2D ^1^H-^15^N HSQC spectra at 25°C over a range of pH values spanning 3.5 to 7.3. The original sample contained 270 μm^15^N-Orb2A PLD and 1.0 mm deuterated acetic acid as a minimal buffer and was titrated with microliter additions of 100 mm sodium carbonate.

##### NMR relaxation measurements.

The dynamics on the ps/ns time scales were probed by measuring the heteronuclear {^1^H}-^15^N NOE of backbone amide groups as the ratio of spectra recorded with and without saturation in an interleaved mode. An 11-s recycling delay was employed. Uncertainties in peak integrals were estimated from the standard deviation of intensities from spectral regions lacking signal and containing only noise. R_1_ρ relaxation rates, which sense dynamic processes on slower µs/ms time scales, were measured by recording two sets of 10 ^1^H-^15^N correlation spectra with relaxation delays at 8, 300, 36, 76, 900, 100, 500, 156, 200, and 700 ms. The R_1_ρ relaxation rates were calculated by least-squares fitting of an exponential decay function to peak integral data, which were obtained using Topspin 4.0.8.

##### Ca^2+^ and Zn^2+^ binding experiments followed by 1D ^1^H NMR.

Two aliquots of ^15^N-labeled Orb2A PLD were lyophilized overnight and then redissolved in 100% D_2_O and 0.20 mm DSS containing 12.5 mm KH_2_PO_4_ + 12.5 mm K_2_HPO_4_, pH* 7.0 or 3.6 mm deuterated sodium acetate (d_3_, 99% atom D, Cambridge Isotope Laboratory) + 21.4 mm deuterated acetic acid (d_4_, >99.5% atom D, Aldrich), pH* 4.0. “pH*” is the pH meter reading without correction for the deuterium isotope effect. The Orb2A PLD concentration was 0.20 mm. H/D exchange was monitored by 1D ^1^H and 2D ^1^H-^15^N HSQC spectra and found to be essentially complete within minutes at pH* 7.0 and within an hour at pH* 4.0. Considering the parameters of Englander and co-workers (54), implemented on the webpage Sphere HX (RRID:SCR_019188), this quick exchange is consistent with a disordered protein containing no or unstable elements of secondary structure. Following exchange, only the aromatic ^1^H of His, Tyr, and Phe are present in the downfield region of the 1D ^1^H NMR spectrum of Orb2A PLD. Changes in these signals were monitored at 25°C following the addition of Ca^2+^ and Zn^2+^ from stock solutions of 100 mm CaCl_2_ (Pancrea) and 100 mm ZnCl_2_ (Fluka) prepared in D_2_O.

##### Binding to RNA oligos.

RNA oligos with sequences ^5′^UUU-UUAU^3′^, the consensus ligand, as well as ^5′^CCCCCGC^3′^, the opposite sequence, were purchased from Integrated DNA Technologies. Following the cleaning of pipettes and surfaces with RNase Zap® (Sigma), they were dissolved as concentrated stock solutions in 50 μl of mQ H_2_O, which had been previously boiled and cooled. RNA stock solutions' concentrations were calculated by UV absorbance at 260 nm and the stocks were incubated on ice until use. Samples containing 200 μm^15^N-labeled Orb2A PLD in either 25 mm K_2_HPO_4_/KH_2_PO_4_ buffer, pH 7.0, or 25 mm deuterated sodium acetate/deuterated acetic acid buffer, pH 4.0, with 0.20 mm DSS and 10% D_2_O/90% mQ H_2_O were prepared and their ^1^H-^15^N HSQC spectra were recorded at 25°C. Then, an RNA oligo was added to the Orb2A PLD sample to give a final molar ratio of 1:1 and another ^1^H-^15^N HSQC spectrum was recorded. Experiments were repeated for each pH and type of RNA oligo. Upon completion, the pH of each sample was measured and found to be 3.98 for the Orb2 + UUUUUAU sample, 3.96 for the Orb2 + CCCCCGC sample, and 6.87 for both samples prepared in potassium phosphate buffer.

##### Bioinformatic analysis.

Disorder predictions for the Orb2A sequence were performed with PONDR-XSL2 and PONDR-XL1-XT (55).

## Data availability

NMR chemical shifts of the Orb2A PLD recorded at pH 4 and at pH 7 have been deposited in the BMRB data bank under file number 50274. The raw NMR data can be requested from D. V. Laurents at dlaurents@iqfr.csic.es. All other data presented and discussed are contained within the article and its supporting material.
