# Supplementary material for: Structural transitions in Orb2 prion-like domain relevant for functional aggregation in memory consolidation
Source: J Biol Chem. 2021 Jan 13;295(52):18122–33. doi: 10.1074/jbc.RA120.015211 (PMC7939463; doi:10.1074/jbc.RA120.015211)
Supplement: Supplementary file 1 [file mmc1.docx]

**Supporting Information**

**Structural transitions in Orb2 prion-like domain relevant for**

**functional aggregation in memory consolidation**

Javier Oroz^1^, Sara S. Félix^2^, Eurico J. Cabrita^2^ and Douglas V. Laurents^1,*^

^1^Instituto de Química-Física Rocasolano, IQFR-CSIC,

Serrano 119, E-28006 Madrid, Spain.

^2^ UCIBIO, Departamento de Química, Faculdade de Ciências e Tecnologia,

Universidade Nova de Lisboa, 2829-516 Caparica, Portugal

*Corresponding Author: dlaurents@iqfr.csic.es

**Contents**:

**Sup. Table 1:** NMR Spectral Parameters

**Sup. Fig. 1:** Orb2 domain and amyloid core structure

**Sup. Fig. 2.** Predicted regions of order and disorder in Orb2A

**Sup. Fig. 3** Full 2D ^1^H-^15^N HSQC spectra of Orb2A PLD with the

Asn/Gln side chain ^1^H_2_-^15^N region.

**Sup. Fig. 4:**  3D ^1^H-^1^H-^15^N NOESY·HSQC correlations evince helical conformations for residues 55-60 of Orb2A PLD at pH 4.0.

**Sup. Fig. 5.** pH induced ^15^N chemical shift changes suggest increased helicity for

Q/H-rich segments at neutral pH.

**Sup. Fig. 6**. ^1^H-^13^C HSQC spectral evince that His residues of Orb2 PLD bind Zn^++^.

**Sup. Fig. 7**. Orb2A PLD His residues do not bind Ca^++^, but do bind Zn^++^ at neutral pH.

**Sup. Fig. 8.** Distinct RNA oligos bind to Orb2A PLD at pH 4 but not at pH 7.

**Sup. Table 1:** NMR Spectral Parameters

| **Experiment** | **Number of Scans** | **Sweep Width (ppm)** | **Matrix** |
| --- | --- | --- | --- |
| *Assignment pH 4.0* | | | |
| 1D ^1^H | 8 | 10 | 32k |
| 2D ^1^H-^15^N HSQC | 4 | 10 ^1^H x 20 ^15^N | 2k x 512 |
| 2D CON^*^ | 4 | 11 ^13^CO x 35 ^15^N | 1k x 512 |
| 3D HNCO | 8 | 10 ^1^H x 20 ^15^N x 11 ^13^C | 2k x 64 x 128 |
| 3D hacacoNcaNCO^*^ | 8 | 11 ^13^CO x 35 ^15^N x 20 ^15^N | 1k x 48 x 96 |
| 3D hacaCOncaNCO^*^ | 8 | 11 ^13^CO x 35 ^15^N x 21 ^13^CO | 1k x 48 x 96 |
| 3D HncocaNH | 8 | 10 ^1^H x 20 ^15^N x 10 ^1^H | 2k x 96 x 48 |
|  |  |  |  |
| 3D CCC...CON^*^ |  | 11 ^13^CO x 35 ^15^N x 60 ^13^C(aliphatic) | 1k x 56 x 96 |
| 3D CBCACON^*^ | 8 | 11 ^13^CO x 35 ^15^N x 70 ^13^C(aliphatic) | 1k x 64 x 80 |
| 3D HNCA | 4 | 11 ^1^H x 20 ^15^N x 25 ^13^C | 2k x 48 x 128 |
| 3D HNHA^§^ | 8 | 11^1^HN x 23 ^15^N x 11 ^1^Hα | 2k x 96 x 48 |
| 3D NOESY·HSQC (t_mix_= 100 ms) | 8 | 10 ^1^H x 20 ^15^N x 10 ^1^H | 2k x 40 x 128 |
| *Assignment pH 7.0 (both before and after adding ZnCl_2_)* | | | |
| 2D ^1^H-^15^N HSQC | 4 | 10 ^1^H x 20 ^15^N | 2k x 512 |
| 3D HNCO | 16 | 10 ^1^H x 20 ^15^N x 11 ^13^C | 2k x 32 x 64 |
| 3D HNCA | 12 | 10 ^1^H x 20 ^15^N x 20 ^15^N | 2k x 64 x 90 |
| 3D CBCAcoNH | 16 | 10 ^1^H x 20 ^15^N x 70 ^13^C | 2k x 32 x 64 |
| *pH titration* | | | |
| 1D ^1^H | 64 | 12 | 2k |
| 2D ^1^H-^15^N HSQC | 8 | 10 ^1^H x 20 ^15^N | 2k x 256 |
| *Spectra recorded at 25ºC, pH 3.5, 4.7, 5.4, 6.1 & 7.3* | | | |

*RNA binding*

| 1D ^1^H | 64 | 12 | 2k |
| --- | --- | --- | --- |
| 2D ^1^H-^15^N HSQC | 4 | 10 ^1^H x 20 ^15^N | 2k x 512 |

*Spectra recorded at 25ºC, pH 4.0 or 7.0 with or without ^5’^UUUUUAU^3’^ or ^5’^CCCCCGC^3’^*

*1D ^1^H NMR to test for Ca^++^ and Zn^++^ binding*

| 1D ^1^H | 64 | 16 | 32k |
| --- | --- | --- | --- |

*Spectra recorded at 25ºC, pH 4.0 or 7.0*

* ^13^C deteccion; processed with in-phase anti-phase (IPAP) virtual decoupling.

§ Recorded to measure ^1^HN-^1^Hα coupling constants.

¶ A delay of 11 seconds between pulses was used. Recorded in interleaved mode.

‡ Ten spectra with delays of 8, 300, 36, 76, 900, 100, 500, 156, 200 and 700 ms were recorded.

**Sup. Fig. 1:** Orb2 domain and amyloid core structure

**
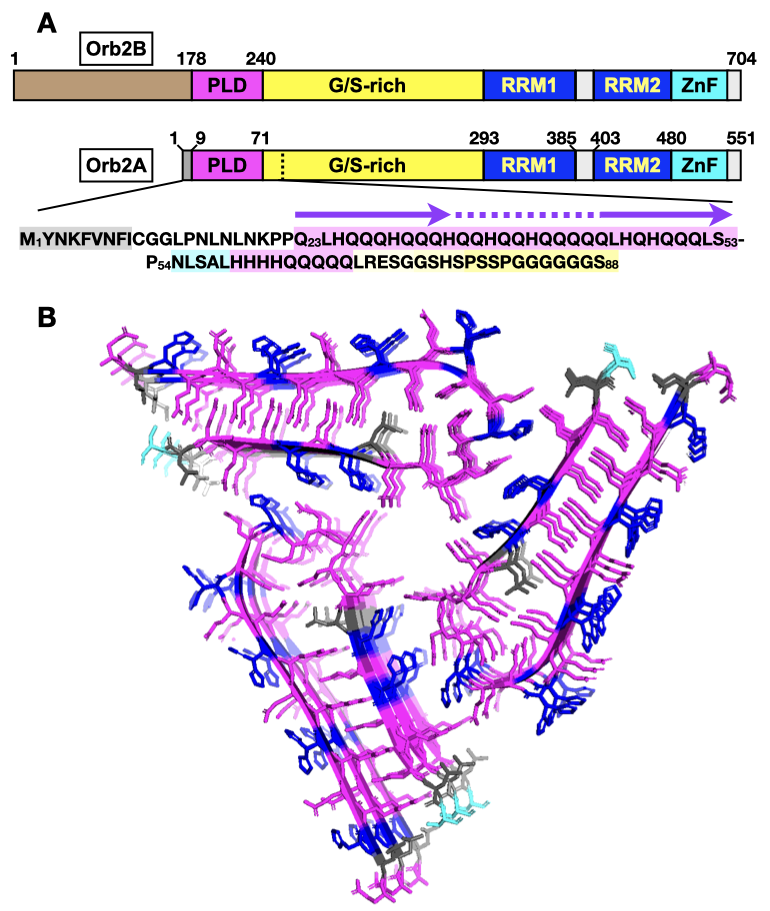
**

**A.** Orb2A and Orb2B differ at the N-terminus (**brown** versus **gray** regions) but both contain a PLD (**magenta**) two RRMs, and a ZZ-type ZnF domain (**turquoise**). The sequence of the OrbA region studied here includes a hydrophobic N-terminal segment (shaded **gray**), a Q/H-rich amyloid-forming segment (shaded light **magenta**), a short segment prone to form α-helix (shaded **blue**), a second modest H·Q-rich segment (shaded light **magenta**), and the beginning of the G/S-rich region (shaded **yellow**). The purple arrows and dotted lines mark residues adopting β-strands and a turn, respectively, in the amyloid core structure shown in panel **B**.

**B**. Structure of the Orb2 amyloid core (**PDB: 6VPS**), solved by cryo-EM (Hervás *et al.,* 2020a). Residue color code: Q = **magenta**, H = **blue**, L = **gray**, S = **cyan**.

**Sup. Fig. 2.** Predicted regions of order and disorder in Orb2A

(Related to **Sup.** **Fig 1A** and the Introduction in the main text)


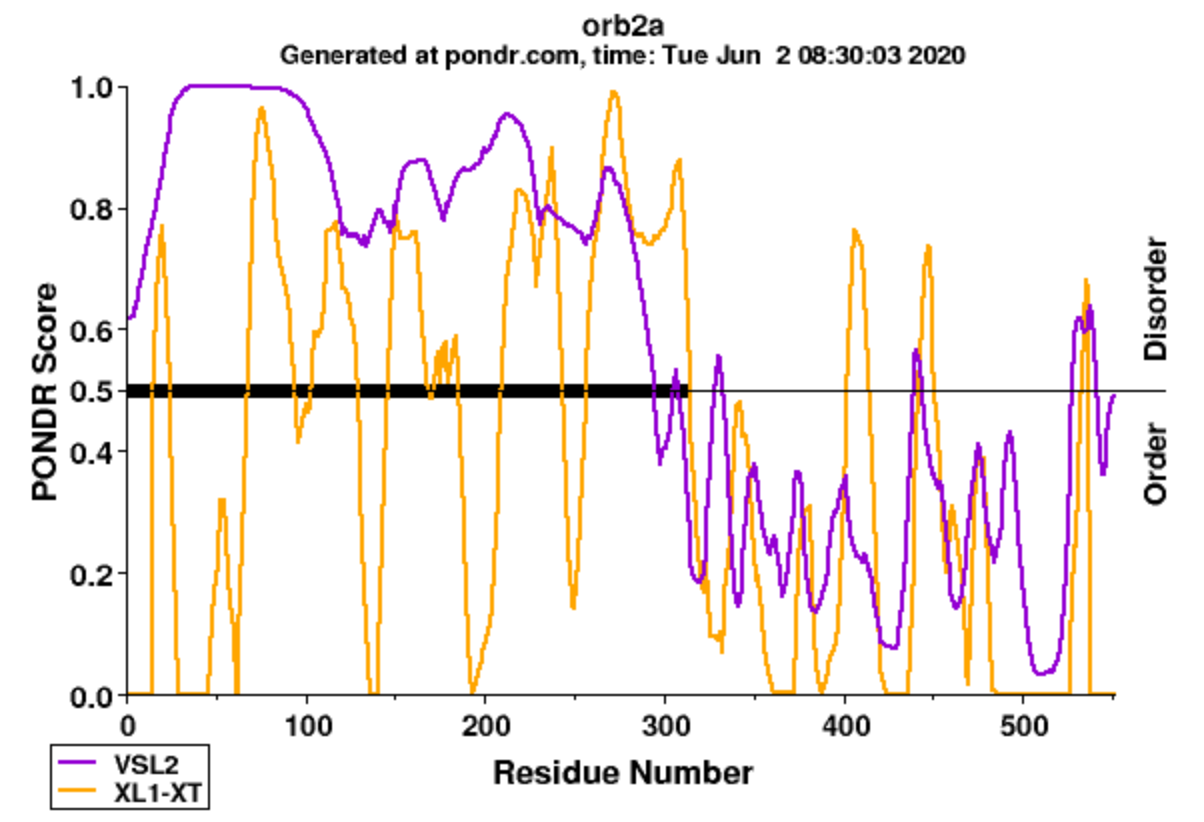


Analysis with the program PONDR predicts that Orb2A’s PLD and G/S-rich regions will be unfolded. The folded RRM and ZnF domains, which begin near residue 300 (as shown in **Sup.** **Fig. 1A**) are correctly predicted to be ordered.

**Sup. Fig. 3** Full 2D ^1^H-^15^N HSQC spectra of Orb2A PLD with the

Asn/Gln side chain ^1^H_2_-^15^N region.

**(**Related to **Figure 1A** and **3A** in the Main Text**)**


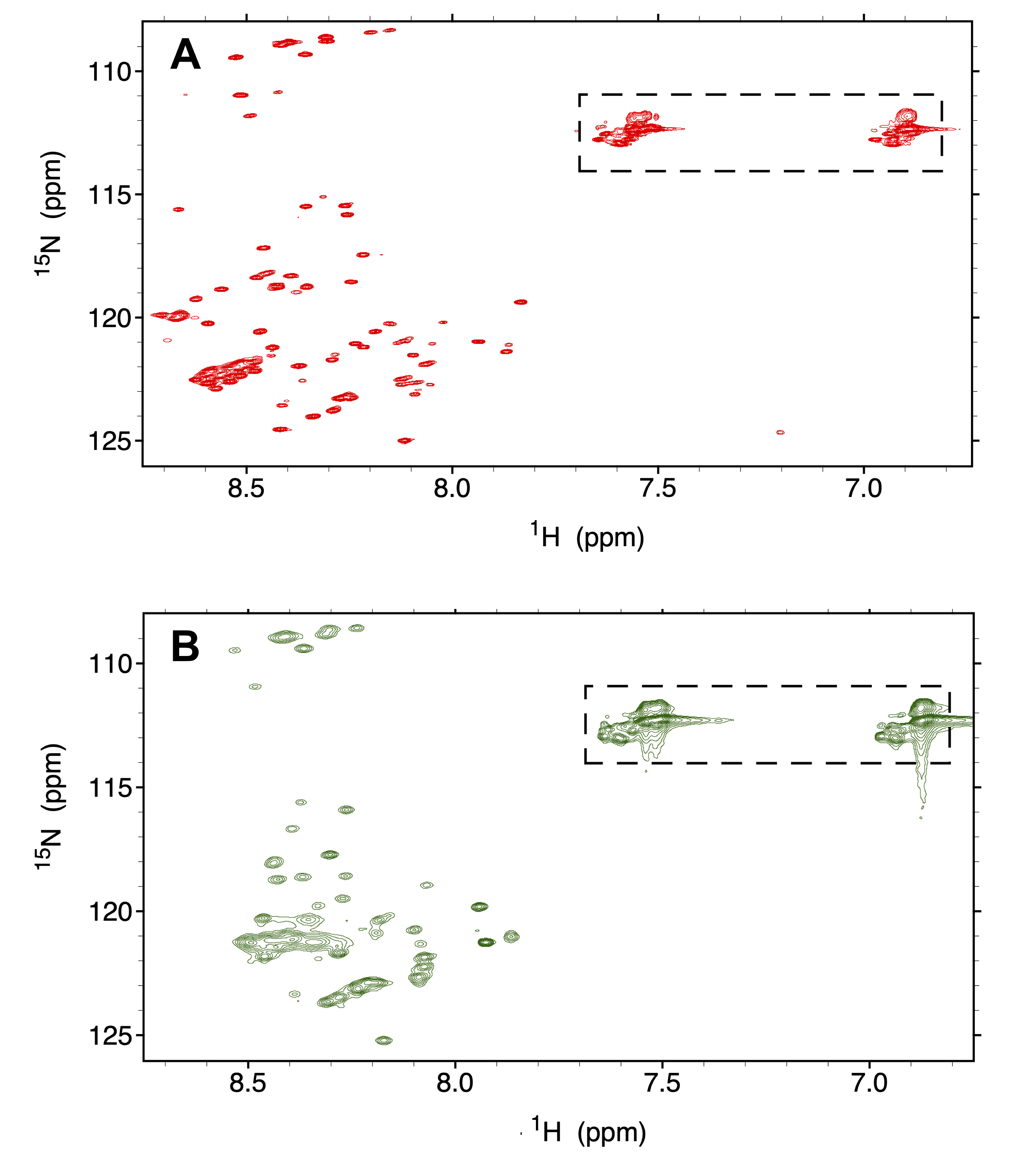


1. 25ºC, pH 4, 1 mM deuterated acetic acid. The Asn/Gln side chain H_2_N- signals are boxed.
2. 25ºC, pH 7, in PBS buffer. The Asn/Gln side chain H_2_N- signals are boxed.

**Sup. Fig. 4:**  3D ^1^H-^1^H-^15^N NOESY·HSQC correlations evince helical conformations for residues 55-60 of Orb2A PLD at pH 4.0. **(**Related to **Figure 2)**


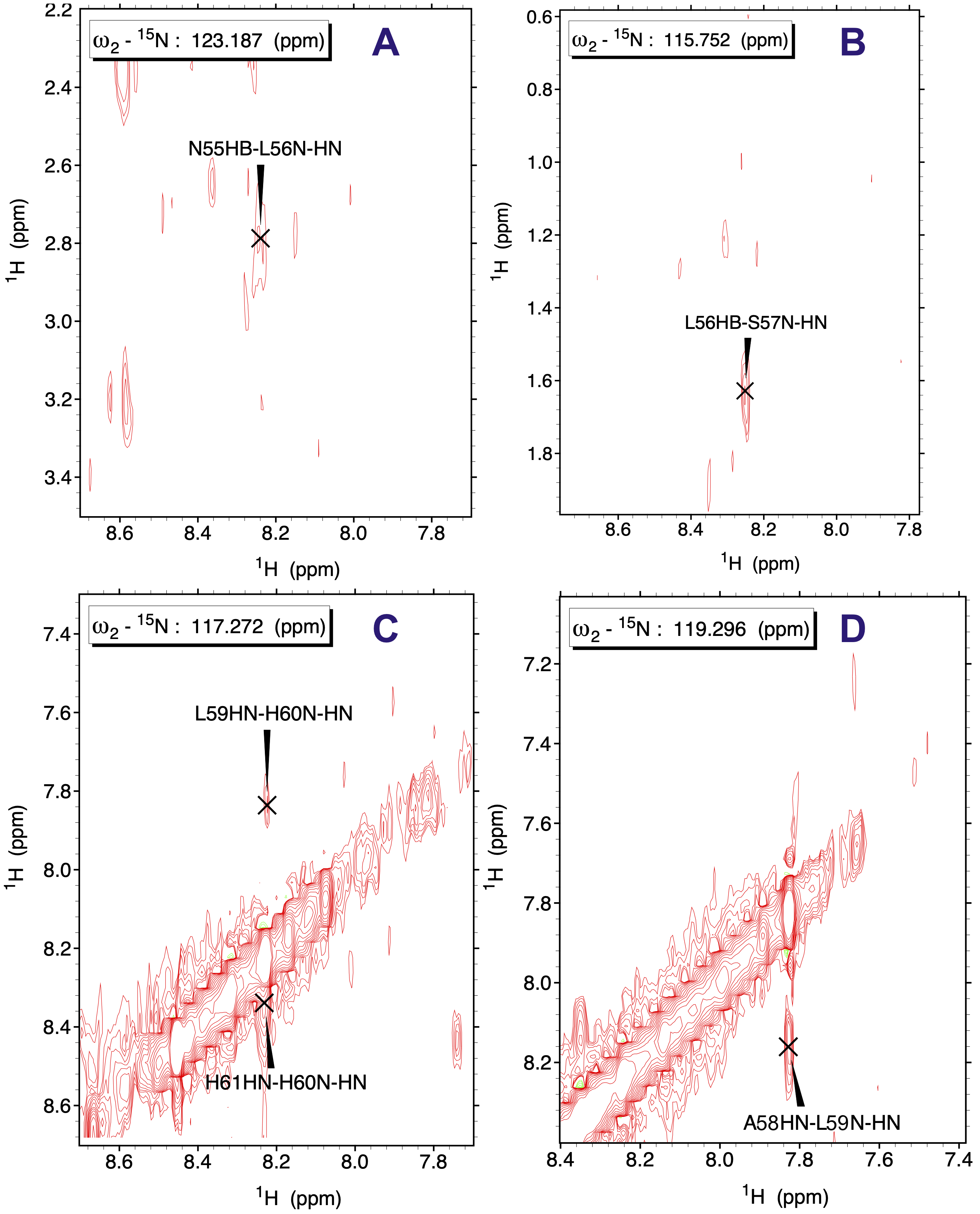


^15^N planes of a 3D ^1^H-^1^H-^15^N NOESY·HSQC spectrum showing representative ^1^Hβ_i_ - ^1^HN_i+1_ (panels **A** & **B**) and ^1^HN_i_ - HN_i+1_ (panels **C** & **D**) NOE correlations which are characteristic of helical conformations. The spectrum recorded with a mixing time of 100 ms at pH 4. In all panels, the multiplication factor between contours is 1.4.

**Sup. Fig. 5.** pH induced ^15^N chemical shift changes suggest increased helicity for

Q/H-rich segments at neutral pH. (Related to **Figure 3** in the Main Text)


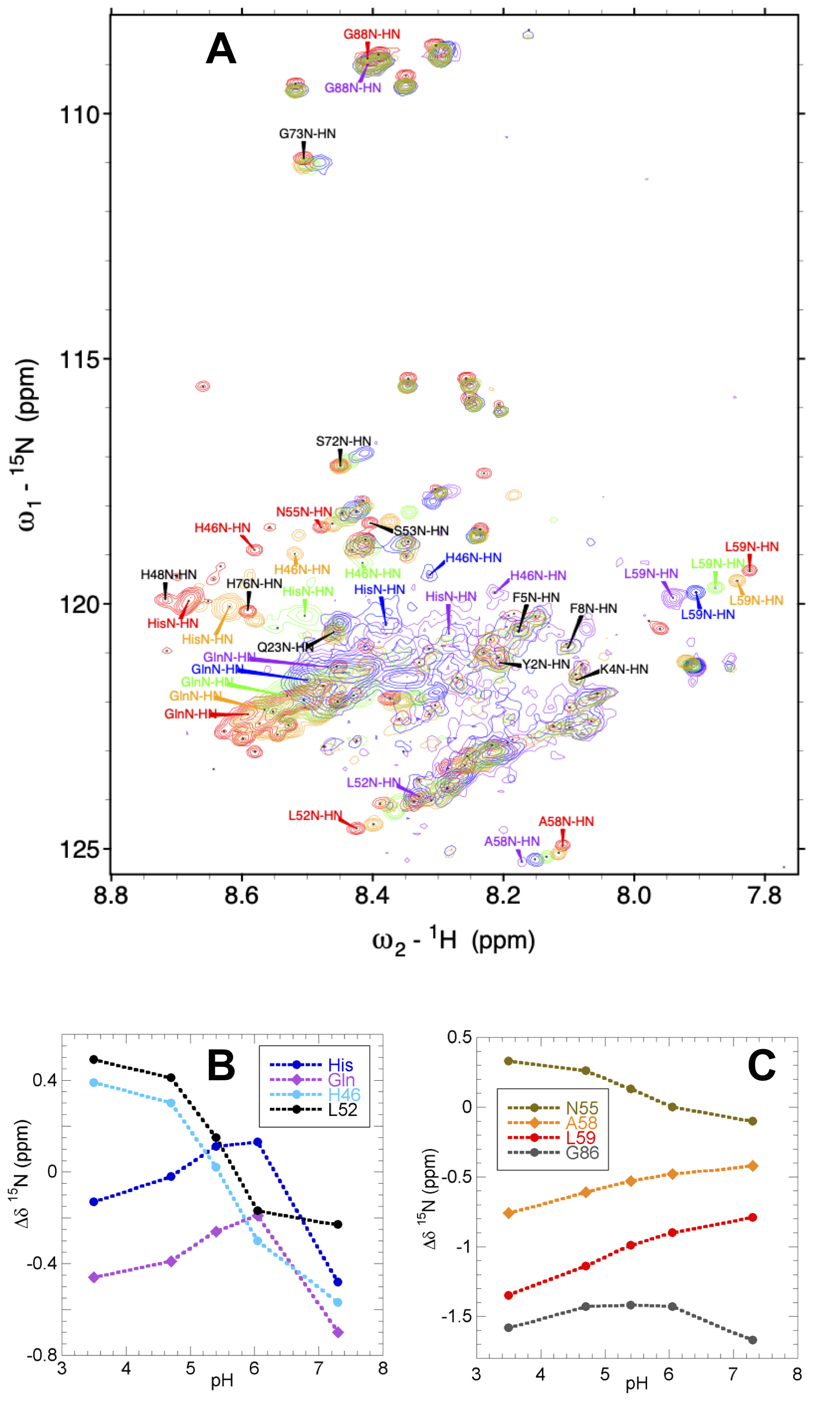


1. The ^1^H-^15^N spectra were recorded at 25ºC and are colored **red for pH 3.5**, **orange for pH 4.7**, **green for pH 5.7**, **blue for pH 6.05** and **purple for pH 7.3**. The relation between Δδ^15^N and pH is plotted for (**B**) representative residues in the Q/H-rich segments, and (**C**) the Gly-rich C-terminal segment and the α-helix spanning residues 55-60.

**Sup. Fig. 6**. ^1^H-^13^C HSQC spectral evince that His residues of Orb2 PLD bind

Zn^++^. (Related to **Figures 4** and **5** in the Main Text)


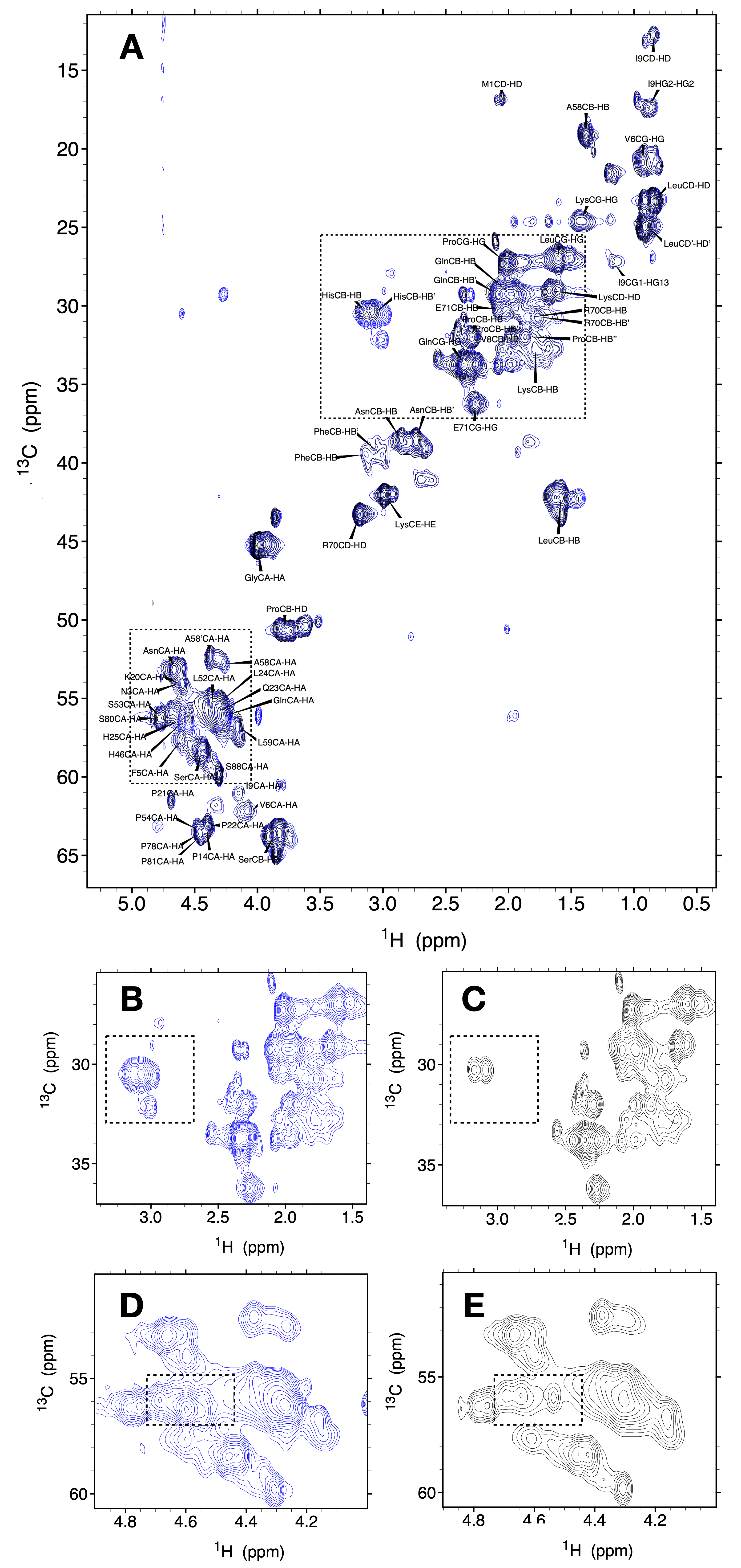


**A**. Complete, assigned ^1^H-^13^C HSQC spectrum at 25ºC, pH 7 in PBS without zinc chloride (**blue**) and with zinc chloride (**black**). Zoomed views of the boxed areas are shown in panels **B**, **C**, **D** & **E**.

**B** & **C**. Zoomed view of panel **A**; the boxed area here highlights the histidine ^1^Hβ-^13^Cβ correlations

before (**B**) and after (**C**) ZnCl_2_ addition.

**D** & **E**. Zoomed view of panel **A**; the boxed zone shows the histidine ^1^Hα-^13^Cα correlations prior to (**D**) and following (**E**) ZnCl_2_ addition.

**Sup. Fig. 7**. Orb2A PLD His residues do not bind Ca^++^, but do bind Zn^++^ at neutral pH.

(Related to **Figures 4** and **5** in the Main Text)


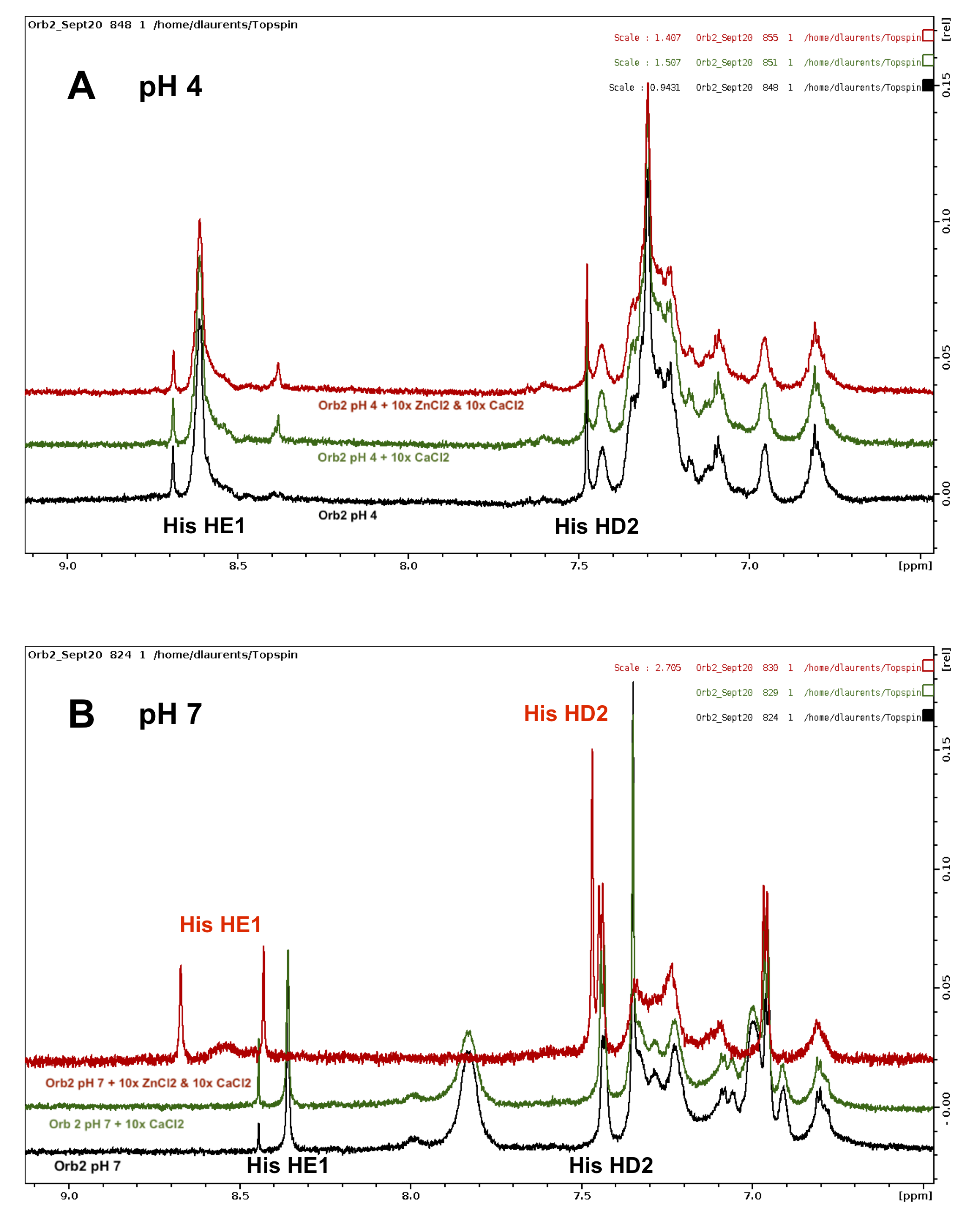


The downfield region 1D ^1^H NMR spectra of 200 μM Orb2A PLD samples (which had been incubated in 100% D_2_O to remove ^1^HN resonances by H/D exchange) features sharp ^1^Hδ2 and ^1^Hε1 signals arising from the twelve His residues and broader signals coming from Phe and Tyr ^1^Hδ, ^1^Hε and ^1^Hζ nuclei.

**A**. At pH 4, 25ºC, Orb2 PLD alone is shown in the **black** trace. No remarkable changes are seen following the addition of CaCl_2_ to a final concentration of 2.0 mM (**green** trace) or following the subsequent addition of ZnCl_2_ to a final concentration of 2.0 mM (**red** trace).

**B**. At pH 7, 25ºC Orb2 PLD alone (**black** trace) or with 10 mM CaCl_2_ (**green** trace) show very similar spectra. Note that the His Hδ2 and ^1^Hε1 signals have shifted relative to pH 4 due to the loss of H^+^. Following the addition of ZnCl_2_ to a final concentration of 2.0 mM, the His Hδ2 and ^1^Hε1 signals show significant changes (**red** trace), evincing that Zn^++^ has bound.

**Sup. Fig. 8.** Distinct RNA oligos bind to Orb2A PLD at pH 4 but not at pH 7.

(Related to **Figures 4** and **5** in the Main Text)


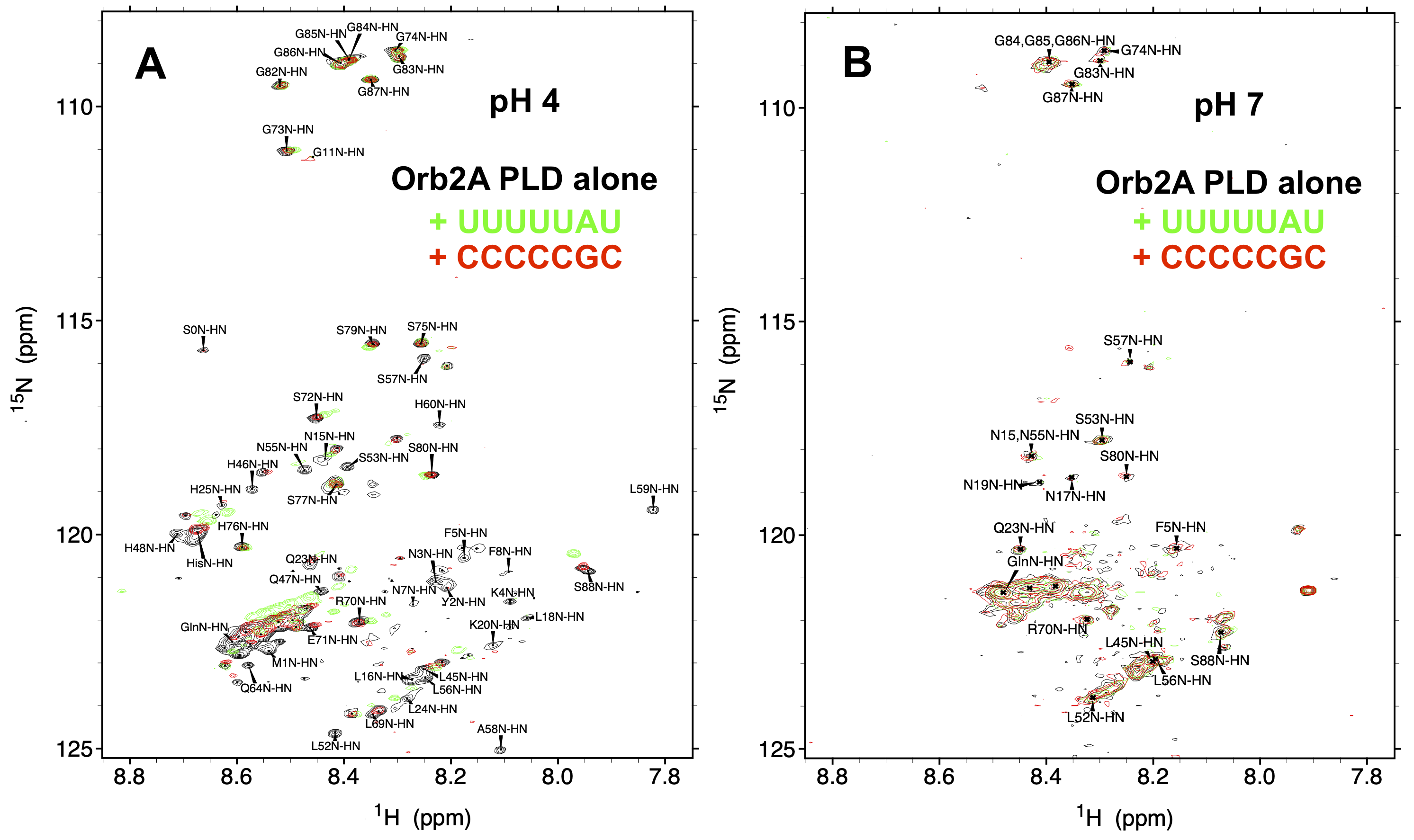


2D ^1^H-^15^N HSQC of Orb2A PLD recorded at 25 ºC at pH 4 (panel **A**) or at pH 7 (panel **B**) in the absence (**black**) or presence of 5’UUUUUAU3’ (**green**) or 5’CCCCCGC3’ (**red**).
